# *In Vivo* Characterization of a Red Light-Activated Vasodilation: A Photobiomodulation Study

**DOI:** 10.3389/fphys.2022.880158

**Published:** 2022-05-02

**Authors:** Agnes Keszler, Brian Lindemer, Grant Broeckel, Dorothee Weihrauch, Yan Gao, Nicole L. Lohr

**Affiliations:** ^1^ Departments of Medicine- Division of Cardiovascular Medicine, Milwaukee, WI, United States; ^2^ Cardiovascular Center, Medical College of Wisconsin, Milwaukee, WI, United States; ^3^ Departments of Plastic Surgery, Medical College of Wisconsin, Milwaukee, WI, United States; ^4^ Institute for Health and Equity- Division of Biostatistics, Milwaukee, WI, United States; ^5^ Clement J Zablocki VA Medical Center, Milwaukee, WI, United States

**Keywords:** vasodilation, endothelium, nitric oxide, red light therapy, photobiomodulation

## Abstract

Nitric oxide dependent vasodilation is an effective mechanism for restoring blood flow to ischemic tissues. Previously, we established an *ex vivo* murine model whereby red light (670 nm) facilitates vasodilation *via* an endothelium derived vasoactive species which contains a functional group that can be reduced to nitric oxide. In the present study we investigated this vasodilator *in vivo* by measuring blood flow with Laser Doppler Perfusion imaging in mice. The vasodilatory nitric oxide precursor was analyzed in plasma and muscle with triiodide-dependent chemiluminescence. First, a 5–10 min irradiation of a 3 cm^2^ area in the hind limb at 670 nm (50 mW/cm^2^) produced optimal vasodilation. The nitric oxide precursor in the irradiated quadriceps tissue decreased significantly from 123 ± 18 pmol/g tissue by both intensity and duration of light treatment to an average of 90 ± 17 pmol/g tissue, while stayed steady (137 ± 21 pmol/g tissue) in unexposed control hindlimb. Second, the blood flow remained elevated 30 min after termination of the light exposure. The nitric oxide precursor content significantly increased by 50% by irradiation then depleted in plasma, while remained stable in the hindlimb muscle. Third, to mimic human peripheral artery disease, an ameroid constrictor was inserted on the proximal femoral artery of mice and caused a significant reduction of flow. Repeated light treatment for 14 days achieved steady and significant increase of perfusion in the constricted limb. Our results strongly support 670 nm light can regulate dilation of conduit vessel by releasing a vasoactive nitric oxide precursor species and may offer a simple home-based therapy in the future to individuals with impaired blood flow in the leg.

## Introduction

The proper physiological function of tissues relies on the timely and appropriate delivery of blood flow. Disruption or derangement of blood flow, either through physical obstruction or impaired responses to cellular agonists, results in ischemia and end organ damage. Among numerous vasodilators the nitric oxide (NO) family represents an effective means to return blood flow to ischemic tissues (Adams et al., 1997; Homer and Wanstall, 2002; Liu et al., 2016). However, the pharmacological use of NO donors is limited by tolerance or medication side effects (Miller et al., 2000; Lundberg et al., 2009; Mohler et al., 2014). Moreover, endogenous production of NO is attenuated in cardiovascular diseases by reduced expression and uncoupling of its associated enzyme endothelial nitric oxide synthase (eNOS). Alternative methods for increasing endogenous NO (Lohr et al., 2009; Shiva and Gladwin, 2009; Keszler et al., 2018; Wajih et al., 2019; Wajih et al., 2021) have identified nitrite, nitrosyl and S-nitrosothiol (RSNO) containing proteins as potential sources. These compounds do not display pharmacological tolerance, nor do they require optimal intracellular oxygen and pH to function. As an alternative, photo relaxation of conduit vessels in the red or near infrared region of the electromagnetic spectrum has gained growing recognition as a non-invasive targeted modality to increase NO supply (Karu et al., 2005; Poyton and Ball, 2011; Ieda et al., 2020; Colombo et al., 2021).

Photobiomodulation (PMB) or low-level laser (light) therapy (LLLT), is the irradiation of tissues with laser or LED in the visible and near infrared range of the electromagnetic spectrum. The cellular mechanisms of photobiomodulation are well described, e. g. proliferation, migration, cytoprotection, inflammation, which contribute to accelerated tissue repair such as wound healing (Freitas and Hamblin, 2016). Irradiation affects a variety of cellular functions with implication of multiple regulatory mechanisms (Karu, 1999). One potential hypothesis for light’s action attributes a central role of complex IV (cytochrome c oxidase, Cco) of the electron transport chain (Karu, 1999; Wong-Riley et al., 2005; Karu T., 2010; Hamblin, 2016), however, numerous other reaction routes may contribute to the general network. Some pathways involve NO which is canonically produced *via* the activity of its synthase enzymes but can also enter the cells from outside by diffusion, or from nitrate and nitrite containing nutrients. The latter can be reduced in many ways (Machha and Schechter, 2011), and specifically in the mitochondrion by the reductase activity of Cco (Poyton and Ball, 2011) or the ubiquionone cycle (Kozlov et al., 1999).

670 nm light facilitates the availability of these NO precursors from their stores (Wong-Riley et al., 2005; Machha and Schechter, 2011), and induces NO release outside of the mitochondria related mechanisms from nitrosylated heme (Lohr et al., 2009; Shiva and Gladwin, 2009), non heme dinitrosyl iron complexes (DNIC) or S-nitrosothiols (RSNO) (Keszler et al., 2018; Wajih et al., 2019; Wajih et al., 2021). Red light synergistically enhances the vasodilatory effect of nitrite (Lohr et al., 2009; Wajih et al., 2019; Wajih et al., 2021) and is capable to liberate NO from nitrosyl and nitroso derivatives (Lohr et al., 2009; Keszler et al., 2018). N-nitroso compounds (RNNO) may also be a putative target of photolytic cleavage to NO, however, mainly at shorter wavelengths, and with a minor relaxing effect (Rodriguez et al., 2003).

Red light treatments reduce the damage observed in traumatic brain injury and stroke models (Hamblin, 2016), as well as cardiac ischemia-reperfusion injury through NO mechanisms (Lohr et al., 2009; Shiva and Gladwin, 2009). Small case studies using red light as a therapy for diabetic wounds (Desmet et al., 2006) and chronic venous ulcers (Caetano et al., 2009) suggest clinical relevance.

In earlier studies (Lohr et al., 2009; Keszler et al., 2017; Keszler et al., 2018; Keszler et al., 2019; Weihrauch et al., 2021) we recognized that 670 nm wavelength is optimal to achieve *ex vivo* dilation of dissected blood vessel and the light effect is not hindered by temperature interference. The vasodilation occurs *via* activation of the NO pathway. Investigation of the underlying mechanism revealed a transferable species secreted from the endothelium which is stable and active *ex vivo* for at least for 30 min, and contains NO moiety, and most probably also free NO. The secretion of the dilatory species is under the control of red light.

Here we examined the stability of the endothelium derived NO precursor in an *in vivo* mouse model by detecting blood flow and measuring the levels of the vasodilatory species in plasma and tissue samples collected at the end point. We optimized the conditions by which 670 nm light would maximize blood flow and determined the stability of the vasoactive NO precursor in plasma and muscle tissue under optimal conditions. Then, in order to mimic the clinical conditions of peripheral artery disease (PAD), we placed an ameroid constrictor, a device containing hygroscopic material to partially occlude the vessel, on the proximal femoral artery of mice. Then measured the arteriolar blood flow reduction and its restoration by red light treatment.

## Materials and Methods

### Materials

All used chemicals were purchased from Sigma-Aldrich (St. Louis, MO), unless otherwise indicated.

### Light Sources

The 670 nm LED lamps with power supply were purchased from Quantum Devices Inc. (Barneveld, WI). The power output was measured with X97 Optometer (Gigahertz Optics, Turkenfeld, Germany). The used parameters are summarized in Table 1.

**TABLE 1:** Photobiomodulation parameters.

Manufacturer	Quantum Devices Inc.
Model	Custom made
Type of emitter	LED
Probe	Fiber optic cord w/90° mirror
Wavelength (nm)	670
Power output	36 W
Power density (mW/cm^2^)	25, 50, 100
Exposure duration (min)	5, 10, 15
Area irradiated (cm^2^)	3, full body
Number of points irradiated	1
Number of treatments	1, 1/day for 14 days
Application technique	2 cm from surface

### Laser Doppler Perfusion Imaging

C57BL/6 mice (26.4 ± 0.4 g, *n* = 10) were purchased from Jackson Laboratories. Power analysis for ANOVA was used to determine the number of animals required. The overall mean difference between the ameroid and control groups was about 170 and the standard deviation was about 210. The correlation coefficient was 0.63. The post-hoc power using a paired t-test with a one-sided 0.05 significance level based on these parameters obtained from the data is about 86.4% to detect a difference between the ameroid and control groups. The other figures are for the exploratory analysis. All experimental procedures and protocols used in this investigation were reviewed and approved by the Animal Care and Use Committee of the Medical College of Wisconsin. Furthermore, all procedures and protocols conformed to the *Guiding Principles in the Care and Use of Animals* of the American Physiologic Society and were in accordance with the *Guide for the Care and Use of Laboratory Animals*. Protocol number is 2,969. For laser doppler measurement mice were anesthetized with 1.25% isoflurane-O_2_. A heat mat was used to control the animal’s temperature during LDI. Since a black background is necessary for proper LDI imaging, the heat mat was placed underneath the imaging mat. Rectal temperature was maintained at 37.0 ± 0.5°C for the duration of the surgery and during LD. The hair on the hindlimb and thigh region of the posterior side of the mouse was removed. Hindlimb perfusion was assessed noninvasively in the plantar foot (index of the overall leg perfusion) before and immediately after red light treatment (670 nm) by Scanning Laser-Doppler (model LDI2-IR; Moor Instruments, Wilmington, DE). For the optimization, the left paw received no light and served as control for each animal, a 3 cm^2^ area of the right paw was treated with 0–100 mW/cm^2^ of 670 nm light for 10 min. Doppler perfusion of the plantar foot was assessed within anatomically defined regions of interest, consisting of the hind paw margins. The change in Doppler Intensity Units (DIU) pre- and post-red-light treatment was first determined in each paw. When examining the stability of the NO precursor vasodilator the whole body of the animal was exposed to light under optimal conditions and DIU was assessed. In the chronic studies the restricted right limb was irradiated under optimal conditions on the days 1, 2, 4, 7, 9, 11 and 14.

### Ozone-Based Chemiluminescence

After completion of the LDI measurement, mice were euthanized by exsanguination and the collected blood was supplemented with N-ethyl-maleimide (NEM, 26 µl of 250 mM/ml blood) and DTPA (5 µl of 50 mM/ml blood), then centrifuged at 4°C, 5,000xg for 5 min. Plasma was collected, snap frozen in liquid nitrogen, and stored at -80°C. Quadriceps tissues were dissected from both hindlimbs, snap frozen in liquid nitrogen, and stored at -80°C. Before analysis tissue samples were homogenized (Feelisch et al., 2002) in ice cold 50 mM phosphate buffer, pH 7.4 containing DTPA (1 mM) and NEM (10 mM). Samples were analyzed with NO analyzer (Sievers Model 280i) equipped with 50 ml purge vessel in a reducing medium prepared daily from KI and I_2_ in glacial acetic acid and double distilled water (Samouilov and Zweier, 1998). Nitrite was removed from the samples with sulfanilamide (100 mM in 2 N HCl, 10% vol/vol). Typically, 300 µl sample with equivalent volume of antifoaming agent was injected into 15 ml reducing medium. The NO precursor species were quantified based on the detector response for known amounts of S-nitrosoglutathione.

### Ameroid Hindlimb Ischemia

To produce hindlimb ischemia, a 0.25 mm ameroid constrictor was surgically placed onto the proximal femoral artery of mice. After induction with 2–3% isoflurane-O_2_, anesthesia was maintained with 1.5% isoflurane-O_2_. Following isolation of the femoral artery from the femoral vein and nerve, an ameroid was placed just proximal to the lateral circumflex femoral artery (Yang et al., 2008). The incision was closed using interrupted 5–0 vicryl sutures.

Since a commercial supply of appropriately sized ameroids were not available, in-house constrictors were used. A stainless steel 18G needle was cut to approximately 1 mm lengths, and then a notch was cut for the artery to make a C shape. Casein was mixed with water to create a paste, the casein was then pressed into the metal ring, and pressure was applied for 24 h at 37°C to remove air bubbles. After 7 days of 5% formaldehyde treatment, to crosslink the casein, the ameroids were evaluated to make sure the casein had filled the ring completely. The ameroids were dried/cured for at least 2 weeks to allow to harden. An approximately 250 µm wide groove was then cut for the artery (Figure 5A).

### Statistics

**FIGURE 1:**
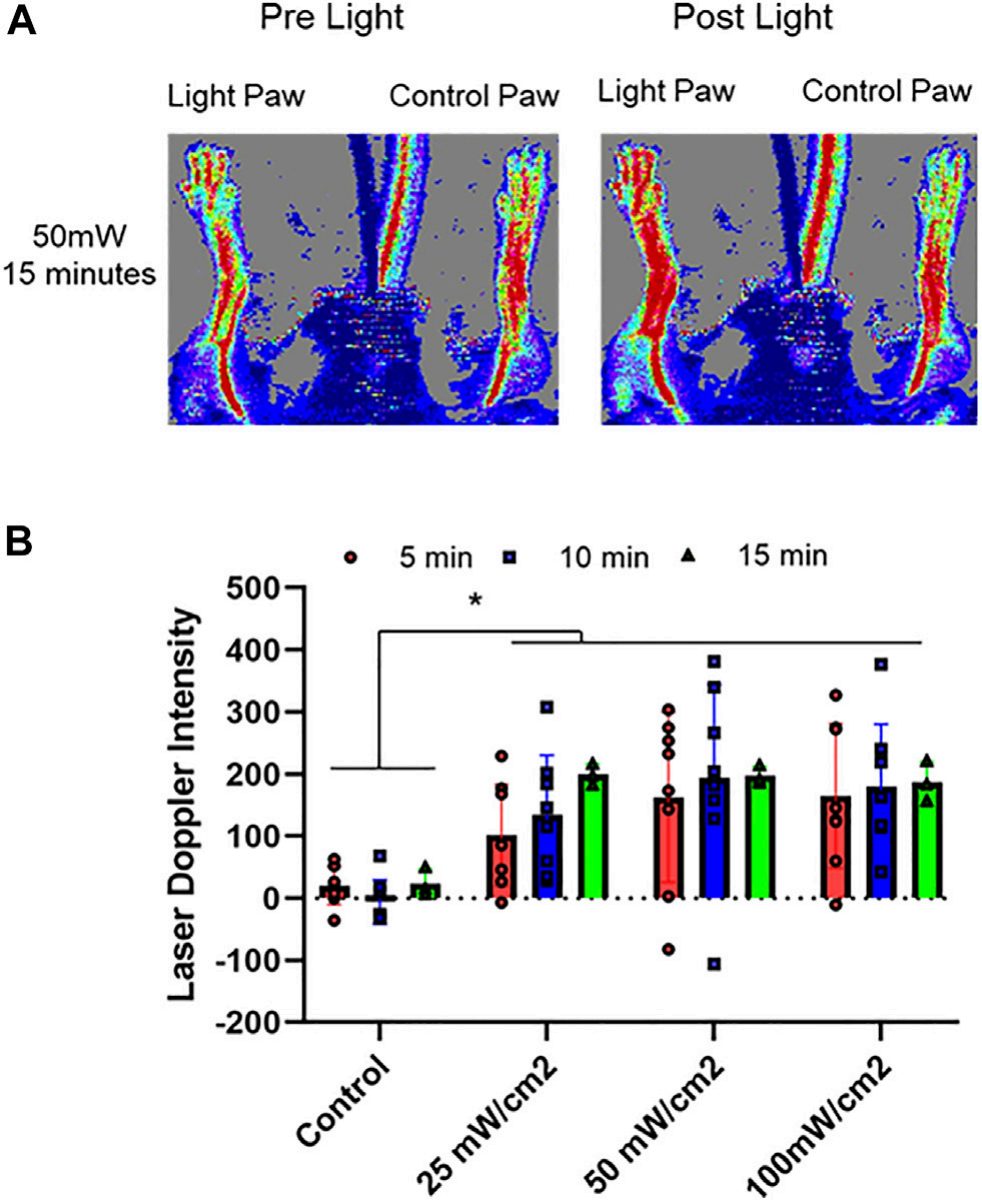
Optimization of vasodilatory effect of red light. 670 nm light was applied at 25, 50, 100 mW/cm^2^ for 5–15 min in hind limb of C57BI/6 mice and blood flow was measured with Laser Doppler Imaging. The flow was measured as a difference between the irradiated limb and the other (control) limb of each animal. **(A)**: Representative image. **(B)**: Quantification of paw perfusion. Mean ± SD is denoted. The Laser Doppler Intensity in irradiated group was significantly greater than that in the control group (*p* < 0.0001). In Friedman’s two-way nonparametric ANOVA, a statistical significance was detected for the exposure duration (*p* = 0.0102), but not for power density (*p* = 0.177).

**FIGURE 2:**
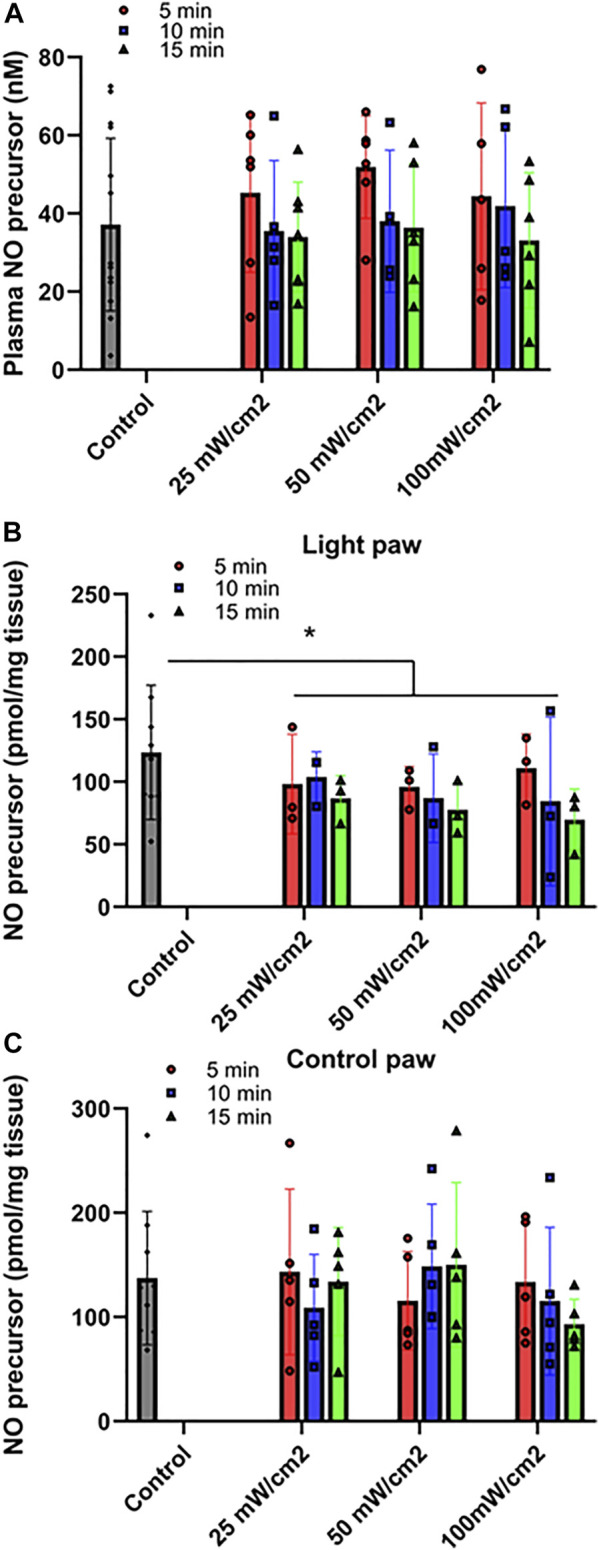
Release of NO precursor species at varying power of red light. Ozone-based chemiluminescence analysis was carried out with triiodide-dependent chemiluminescence in plasma **(A)**, Quadriceps muscle treated with light **(B**) or control limb **(C)**. Mean ± SD is denoted. **(A)**: No statistical significance was detected between the irradiated and control group (*p* = 0.6108). **(B)**: The difference is significant between the irradiated and control groups (*p* = 0.0280). No statistical significance was detected for either power density or exposure durations (*p* = 0.7932 and 0.2902, respectively). **(C)**: No statistical significance was detected between the irradiated and control group (P-value: 0.6323).

**FIGURE 3:**
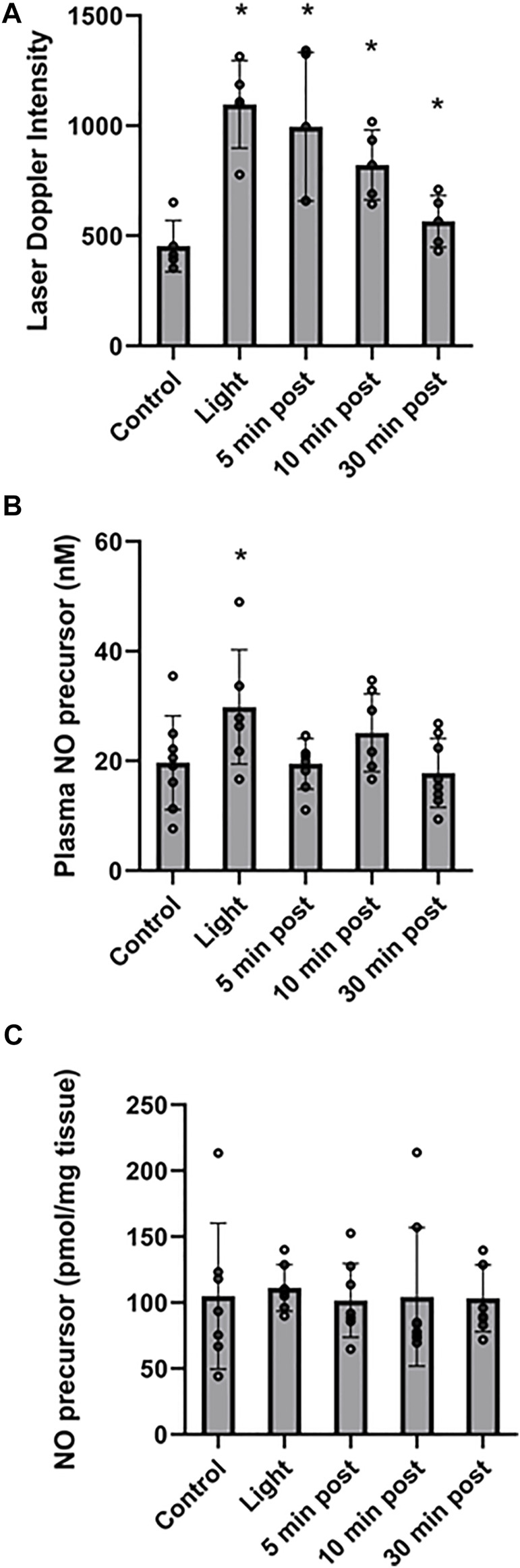
Stability of red light-dependent vasodilator. 670 nm light was applied at 50 mW/cm^2^ for 10 min on entire body of C57BI/6 mice with and the vasodilation was assessed at 0, 5, 10, and 30 min after finishing the irradiation. **(A)**: Blood flow measured with Laser Doppler Imaging. **(B, C)**: The NO-derived vasodilator was measured with ozone-based chemiluminescence in plasma **(B)**, and right quadriceps tissue **(C)**. Mean ± SD is denoted. **(A)**: The difference is significant between each light treatment group compared to the control group (The adjusted P-values by the step-down Bonferroni procedure are 0.0013, 0.0052, 0.0011, and 0.0711, respectively). **(B)**: The overall P-value was 0.0249. Dunnett’s multiple comparison test suggested the “Light” group demonstrated a significant difference compared to the control group (adjusted *p* = 0.0469). The other three light treatments groups did not demonstrate a significant difference. **(C)**: There was no significant difference between the irradiated and control groups (overall *p* = 0.987).

**FIGURE 4:**
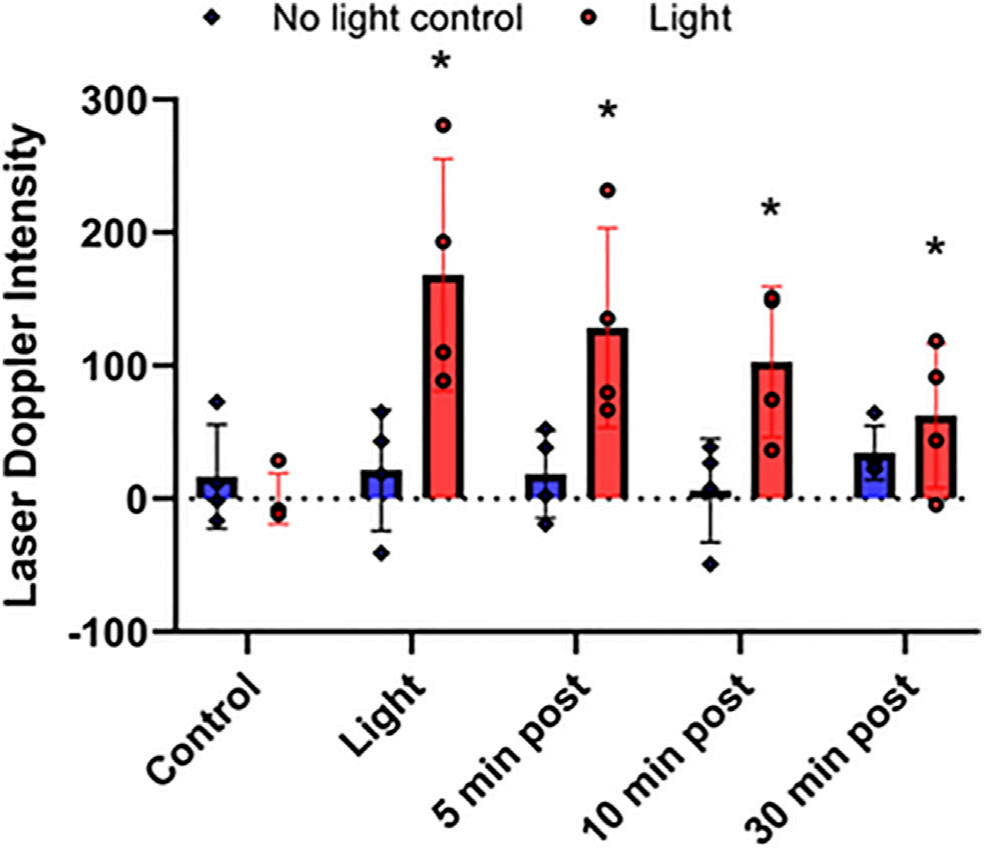
Stability of red light-dependent vasodilator. 670 nm light was applied at 50 mW/cm^2^ for 10 min on right limb of C57BI/6 mice with blood flow was measured with Laser Doppler Imaging. Difference between the irradiated and non-irradiated limbs. Vasodilation was assessed at 0, 5, 10, and 30 min after finishing the irradiation. Mean ± SD is denoted. There was no significant difference in the “No light control” groups (the adjusted P-value by the step-down Bonferroni procedure is 0.3989). In the “Light” group for each time point, there was a significant difference compared to control (adjusted *p* = 0.0189, 0.0189, 0.0330, and 0.0017, respectively).

**FIGURE 5:**
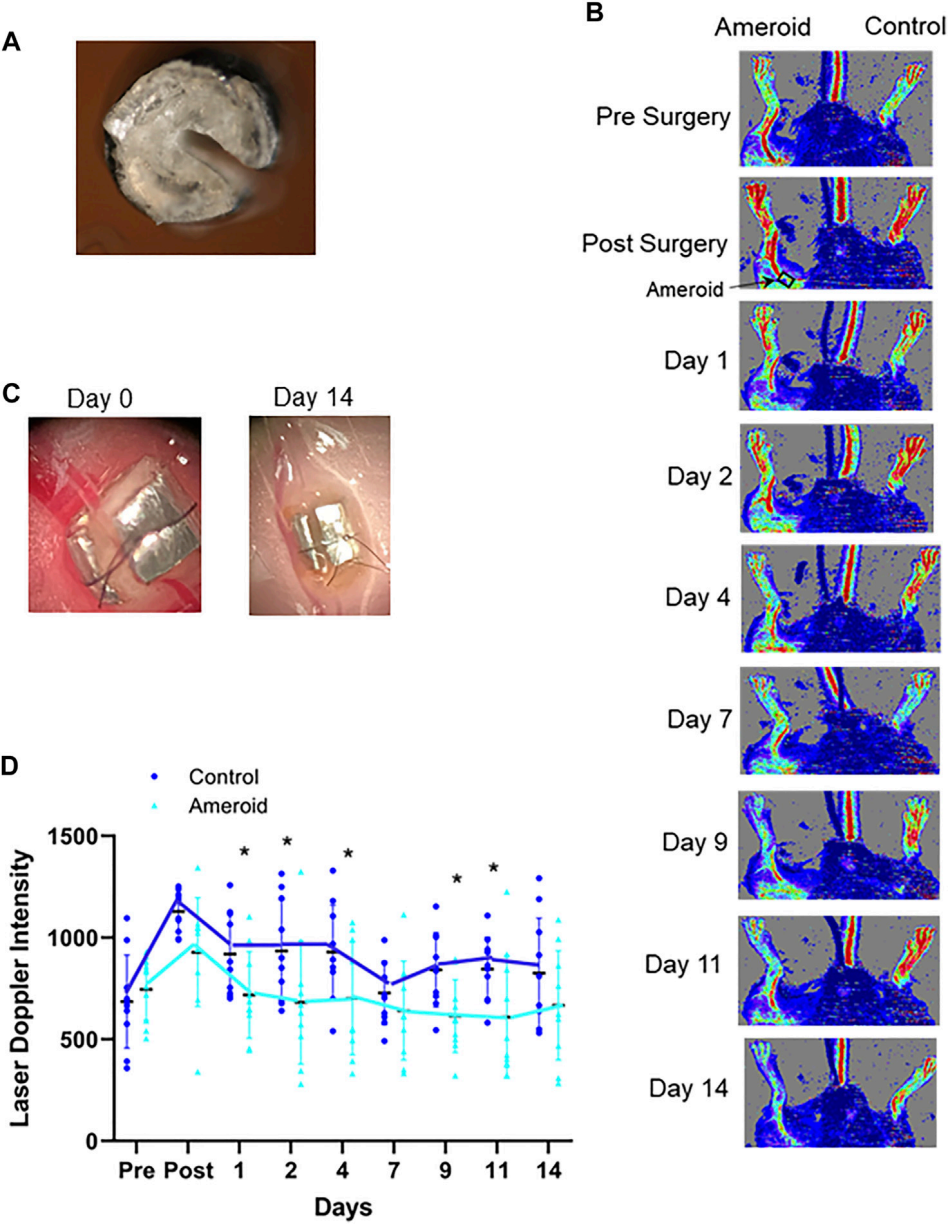
Ameroid constrictor to mimic hindlimb ischemia. **(A, C)**: A representative 0.25 mm internal diameter ameroid constrictor designed, constructed, and implanted on the proximal femoral artery by our laboratory. **(B)**: Laser Doppler Images of C57Bl/6 mice treated with an ameroid in the femoral artery and the contralateral control. **(D)**: Blood flow measured with Laser Doppler Imaging. Mean ± SD is denoted. The adjusted P-values by the step-down Bonferroni procedure between control and ameroid are *p* = 1, 0.162, 0.00178, 0,00101, 2.37 × 10^-8^, 0.932, 0.000202, 0.00868, and 0.289, respectively.

**FIGURE 6:**
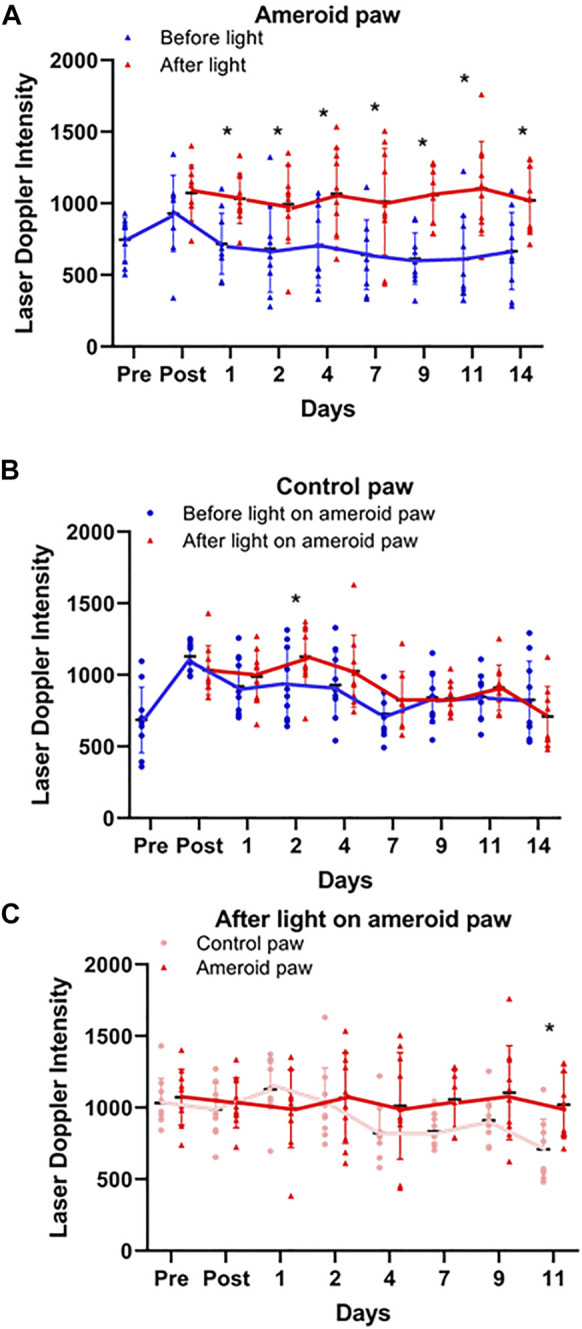
Light effect on ameroid hindlimb ischemia. Doppler perfusion of the plantar foot was assessed pre and post-surgical implantation of the ameroid and for 14 days post-surgery immediately before and after light treatment. **(A–C)** Blood Flow measured by Laser Doppler Imaging before and after 670 nm light on ameroid paw: Ameroid paw **(A)**, Control paw **(B)**, and comparison of both paws after irradiation **(C)**. Mean ± SD is denoted. The adjusted P-values by the step-down Bonferroni procedure between control and ameroid are *p* = 0.165, 3.70 × 10^-15^, 1.25 × 10^-9^, 6.30 × 10^-14^, 1.99 × 10^-5^, 2.28 × 10^-4^, 9.24 × 10^-18^, and 2.57 × 10^-4^, respectively **(A)**, 0.222, 0.146, 0.0284, 0.421, 0.406, 1, 1, and 0.406, respectively **(B)** or control and ameroid 1, 1, 0.343, 1, 0.289, 0.107, 0.136, and 0.0143**(C)**, respectively.

For Figures 1–3, a one-way ANOVA was conducted to assess the impact of light *vs*. the control group on the outcome. A binary variable was constructed to identify whether the mice came from the control or light group (0 *vs*. 1). The control group refers to the mice with no light treatment. The intervention group refers to the mice with at least one kind of light treatment whose power density or exposure duration varies. For Figures 1, 2, if the intervention group demonstrated significant impact, a two-way ANOVA or Friedman’s two-way nonparametric ANOVA was conducted to assess the outcome difference among the light treatment groups, where the power density (3 levels) and exposure durations (3 levels) were considered as two factors. Tukey’s multiple comparison test was conducted for the pairwise comparisons of light treatments while controlling the family-wise Type I error rate. For Figures 3B,C, no power density was considered. A one-way ANOVA was conducted to assess the impact of the four light treatments (0, 5, 10, 30 min exposure duration) *vs*. the control group on the outcome. Dunnett’s multiple comparison test was used to compare each treatment group against the control group. For Figure 3A, Figures 4–6, the mice were repeatedly measured over time. The Generalized Estimating Equations (GEE) method developed by Liang and Zeger (Liang and Zeger, 1986), was employed to account for the correlation over time within mice. The adjusted P-values by the step-down Bonferroni procedure (Holm) was conducted over all the obtained raw P-values to control the family-wise error rate of a two-sided 0.1 level. The step-down Bonferroni procedure corresponds to the methods of Holm (1979) and Shaffer (1986). Holm method is a step-down procedure that increases multiple comparisons’ statistical power. It ranks all the observed/raw *p*-values from the smallest to the largest and guarantees that a *p*-value is never declared significant unless all smaller *p*-values are declared significant (Holm, 1979; Shaffer, 1986). All analyses were performed using SAS^®^ 9.4 (SAS Institute Inc., Cary NC). Sample size used for each experiment are summarized in the Supplementary Material S1.

## Results

### Optimization of Vasodilatory Effect of 670 nm Light

Our previous *ex vivo* studies demonstrate that red light (670 nm) controls vasodilation by secreting a stable nitric oxide precursor from the endothelium, which can be reduced to NO (Keszler et al., 2017; Keszler et al., 2018; Keszler et al., 2019; Weihrauch et al., 2021). Here we tested the functional significance of these findings.

First, we determined the conditions whereby red light would maximize blood flow. (Figures 1, 2). C57Bl6 mice received 670 nm light to the thigh of the right leg for 5–15 min in intensity ranges from 25 mW/cm^2^-100 mW/cm^2^. We found a significant increase in paw blood flow (Figures 1A,B) even after 5 min of the lowest dose light treatment and this increase achieved a clear saturation pattern starting at 25 mW/cm^2^, 15 min. Longer treatment resulted in significantly higher blood flow, although the differences between the various light doses did not reach significance. Based on these experiments, a light intensity of 50 mW/cm^2^ for 5–10 min (fluence of 15, and 30 J/cm^2^, respectively) appears to maximize hindlimb blood flow. We examined the NO precursor levels in the plasma of exsanguinated mice after each LDI measurement (Figure 2A). The values did not show significant change. Since muscle tissue contains nitroso species, we examined whether the light irradiation would impact the levels of NO precursor. Immediately after irradiation was completed, quadriceps muscles from the illuminated hindlimb and the unexposed control hindlimb were dissected for triiodide-dependent chemiluminescence measurements. All light treatment conditions exhibited a significant decrease from 123 ± 18 pmol/g tissue in the control limb to an average value of 90 ± 17 pmol/g tissue after light treatment, while in the muscle tissue of unexposed controls their levels (137 ± 21 pmol/g tissue) did not change (Figures 2B,C). The decreasing trend in the light exposed area *vs*. control hindlimb suggests a moderate light effect.

### Stability of the 670 nm Light Dependent Vasodilator

Recently we found that the NO precursor vasodilatory substance is stable at least for 30 min within the bath solution during pressure myography of murine facialis arteries and is capable to stimulate further vasodilation (Weihrauch et al., 2021). Here we examined the *in vivo* stability of the NO precursor. To measure the NO precursor levels in plasma, all blood from mice should have been collected. When the irradiation was targeted to the thigh, the *in situ* released NO precursor became diluted in the circulation and made it difficult to follow the changes (Figure 2A). Therefore, in the stability experiments the animals were challenged to entire body irradiation with optimal light dose (at 50 mW/cm^2^ for 10 min). The blood flow measured with LDI increased 2.4-fold (from 453 ± 45 DIU to 1,097 ± 75 DIU) by light exposure, and at 30 min post illumination it remained 1.4-times higher (634 ± 51 DIU) than the control level (Figure 3A). Correspondingly, in the plasma samples the NO precursor levels significantly increased by 50% *vs*. control (from 19.7 ± 3.0 nM to 29.9 ± 3.9 nM) immediately after light exposure followed by a decrease to 19.5 ± 1.6 nM already at 5 min after the irradiation stopped and did not gain a significant difference from control or each other during the post exposure time (Figure 3B). Meanwhile, the NO precursor concentration in tissue samples remained steady (105 ± 13 pmol/g tissue in average) during the procedure (Figure 3C). These findings suggest that the vasoactive substance accumulates in the plasma by light exposure, then quickly depletes and is carried over to the tissue where it may replenish the NO stores. Importantly, when LDI experiments were repeated with light exposure to the right limb (Figure 4), blood flow measured as a difference between irradiated and not irradiated paws showed a similar trend to our experiments with entire body irradiation (Figure 3A).

### Effect of 670 nm Light on Ameroid Hind Limb Ischemia

To imitate the clinical conditions caused by human PAD we designed, constructed, and implanted an ameroid constrictor (Figures 5A,C) onto the femoral artery of mice. LDI analysis was performed pre- and post-surgery, and on the days of light treatment both before and after light exposure to determine the effect of light mediated vasodilation in the setting of impaired blood flow (Figures 5B,D). Before application of light, a significant (*p* ≤ 0.03) reduction in flow was detected in the ameroid constricted paw compared to the control throughout the experiment (Figure 5D). Next, to ameliorate blood flow restriction on hind limb ischemic mouse model, we applied red light to the constricted paw (Figure 6 and Supplementary Material S2) under the previously optimized conditions (Figure 1B). Chronic occlusion was continued for 14 days, and the restricted paw was irradiated on the day of 1, 2, 4, 7, 9, 11 and 14. Blood flow significantly increased in the constricted paw after each application of red light and was maintained during the entire investigation (Figure 6A). The light treatment reestablished the blood flow in the constricted paw to the same level as the control paw (Figure 6C). At the same time, there was no change in the perfusion of the control paw, which was neither constricted nor irradiated (Figure 6B).

## Discussion

Previously we found that the *ex vivo* vasodilatory effect of red light (Keszler et al., 2017; Keszler et al., 2019; Weihrauch et al., 2021) is optimal at 670 nm wavelength and occurs *via* an endothelium derived transferable NO precursor. This precursor is stable and active at least for 30 min under physiological conditions. The long half-life of NO precursors in the bath was explained by their presence in microvesicles which subsequently exit the cell by electromagnetic energy (Weihrauch et al., 2021). Our current study extended the *ex vivo* investigations to *in vivo* conditions and convincingly established that 1) the light treatment is optimal at an intensity of 50 mW/cm^2^ for 5–10 min (Figure 1), 2) the NO precursor vasodilator is active and stable for 30 min (Figure 3A), 3) accumulates in the plasma during light exposure, and is quickly drained when the irradiation is finished (Figure 3B). Finally, we mimicked chronic peripheral artery disease by designing and implanting an ameroid constrictor onto the hind limb femoral artery of mice (Figure 5) and restored the restricted blood flow by application of 670 nm light (Figure 6).

Light mediated vasodilation was initially described by Robert Furchgott when UV and blue light induced relaxation of rabbit aorta (Furchgott et al., 1961). Since then, similar studies have shifted to longer wavelengths where the tissue penetration is deeper, and the radiation does not produce harmful side-effects. Most animal studies, *ex vivo* and *in vivo,* focus on wound healing and angiogenesis, while only a minor part targets microcirculation or blood pressure (Colombo et al., 2021). *Ex vivo* light therapy conducted on porcine coronary arteries at 670 and 680 nm wavelengths resulted in a dose dependent relaxation, which was blunted by addition of cPTIO or ODQ suggesting NO-dependent mechanism (Plass et al., 2012). These results are consistent with our previous findings on murine facialis arteries (Keszler et al., 2017). Spontaneously hypertensive rats subjected to *in vivo* irradiation at 660 nm at six different body points, and 69% of them showed a considerable decrease of systolic arterial pressure, accompanied with an increased total serum NO (nitrite plus nitrate) level (Buzinari et al., 2020). In addition, human applications of PMB research on mitigation of endothelial dysfunction are available both in the far red and near infrared regions (Colombo et al., 2021). Application of 890 nm laser for 30 min on the forearm of volunteer human subjects resulted in an increase of total serum NO content of locally drawn venous blood until 5 min in the treatment followed by a drop during the examined period (Mitchell and Mack, 2013). This pattern is similar to what we found in plasma NO precursor levels when mice received full body exposure of 670 nm LED light.

The mechanism of PMB is complex and highly dependent on the applied wavelength and power (Karu, 1999; Colombo et al., 2021; George et al., 2018). Photo acceptor molecules that absorb specific wavelengths of light participate in regulation of metabolic pathways, therefore the action of light is categorized with primary (direct photochemical) and secondary (downstream) effects. Primary targets including mitochondria, various heme proteins and S-nitrosothiols upon activated trigger signaling pathways leading to alteration of physiological events. The mitochondrion features both categories, since photoactivation of the electron transport chain complexes influences the organelle’s ATP production, calcium flux, or reactive oxygen species levels (Karu T. I., 2010; Amaroli et al., 2021). Noteworthy, that energy in the near infrared region is also absorbed by intracellular water layers (George et al., 2018). Further, photo acceptors outside the mitochondria such as nitrosylated iron complexes and nitroso compounds are also critical transmitters of light mediated physiological events.

According to our earlier proposed mechanism (Weihrauch et al., 2021) 670 nm light induces endothelium derived vesicle formation and subsequent exocytosis. The vesicles are vasoactive and contain NO precursors. Previous observation suggests the most probable vasoactive NO precursor is RSNO (Keszler et al., 2019), however, DNIC is also a plausible candidate (Keszler et al., 2018; Keszler et al., 2019), while RNNO compounds and nitrosylated cytoglobin (Zweier and Ilangovan, 2020) cannot be excluded either. These potential NO precursors were all taken into consideration and quantified with triiodide dependent chemiluminescence without discriminating among them with specific reagents. The NO species unrelated to the endothelium, such as nitrosyl hemoglobin (HbNO) and S-nitroso hemoglobin (Hb-SNO) were not considered relevant in the *ex vivo* model, hence RBCs were not analyzed. However, participation of species outside the endothelium cannot be excluded from the overall mechanism.

The reported findings support advancing red light as a non-invasive vasodilator, however some experimental limitations in this study remain. The limited plasma volume from mice required exsanguination, therefore locally drawn blood could not be collected to detect sufficient amounts of the NO precursor. Since NO has been implicated as a mediator of red-light stimulated angiogenesis, the relative contribution of angiogenesis to the present observations must be considered. Murine hindlimb angiogenesis is well described 7 days after femoral artery excision and severe ischemia (Lohr et al., 2013). The ameroid model used in this study does not abolish blood flow, therefore the angiogenic stimulus of ischemia is lessened. Therefore, we proffer the acute changes in blood flow at early timepoints (less than 7 days) do not coincide with angiogenesis. The contribution of angiogenesis to blood flow changes at later timepoints are less certain, and we intend to explore the potential role of angiogenesis in ameroid ischemia in the future. Penetration of the light into tissues should be considered if practical therapies are to be developed. The penetration depth of the employed wavelength is reported to be 4–5 mm (Ash et al., 2017) at 10 J/cm^2^, but our Monte Carlo analysis (unpublished data) gave 10% energy retention at 15–20 mm at the applied 22.5 J/cm^2^. This depth range can interact with dermal vasculature. Moreover, this study supports that red light irradiation secretes a vasodilator into the bloodstream. This finding is important when discussing the penetration depth because a humoral factor has the potential to indirectly stimulate vascular function at greater depths. An additional consideration for any therapeutic device design should include an assessment of heat. The LED array was set 1 cm above the target to ensure any heat generation was dissipated by ventilation. Temperature was monitored during the procedure and did not show an increase. Previously we have confirmed that red light illumination does not significantly increase temperature and 37°C temperature only caused less than 1.1% dilation under *ex vivo* conditions (Keszler et al., 2018). Nevertheless, our overall results support the hypothesis that 670 nm light effectively increases vasodilation by generating a NO-dependent vasodilator in deep tissues, and these findings may serve as a basis for future therapies.

Investigating mechanisms which improve cardiovascular function is vital to developing future therapies for vascular diseases. Low-level red-light therapy increases NO bioavailability locally and most efficiently when enzymatic NO production is compromised during ischemia. PAD is a highly prevalent disease affecting over eight million Americans and generates a high ischemic burden secondary to occlusive arterial disease and endothelial dysfunction (Matsushita et al., 2019). Our murine ameroid constrictor model proved to be a good means to investigate the mitigation of symptoms of this disease. The research presented here focuses on physiological aspects of light delivery and concludes that 670 nm light treatment increases perfusion. Since non-invasive therapies for PAD patients are limited, red-light therapy may offer an effective treatment of individuals with impaired blood flow and tissue ischemia.

## Data Availability

The raw data supporting the conclusions of this article will be made available by the authors, without undue reservation.

## Supplementary Material

The Supplementary Material for this article can be found online at: https://www.frontiersin.org/articles/10.3389/fphys.2022.880158/full#supplementary-material

Supplementary Figure S1 Sample size.

Supplementary Figure S2 Representative image of the LDI measurement using ameroid constrictor. The animals received the optimal duration and intensity of 670 nm light for 14 days. Doppler perfusion of the plantar foot was assessed within anatomically defined regions of interest, consisting of the hind paw margins, immediately before and after light treatment.

## Abbreviations

Cco, cytochrome c oxidase; PMB, photobiomodulation; cPTIO, 2-(4-carboxyphenyl)-4,5-dihydro-4,4,5,5-tetramethyl-1H-imidazolyl-1-oxy-3-oxide; DNIC, dinitrosyl iron complexes; DIU, Doppler Intensity Units; DTPA, diethylenetriamine pentaacetic acid; eNOS, endothelial nitric oxide synthase; HbNO, nitrosyl hemoglobin; Hb-SNO, S-nitroso hemoglobin; LDI, Laser Doppler Perfusion Imaging; LLLT, low level light therapy; LED, light-emitting diode; NEM, N-ethyl-maleimide; ODQ, 1H-[1,2,4]Oxadiazolo[4,3-a]quinoxalin-1-one; PMB, photobiomodulation; PAD, peripheral artery disease; RSNO, S-nitrosothiols; RNNO, N-nitroso compounds; RBC, red blood cells.

## References

[B1] Adams M. R. McCredie R. Jessup W. Robinson J. Sullivan D. Celermajer D. S. (1997). Oral L-Arginine Improves Endothelium-Dependent Dilatation and Reduces Monocyte Adhesion to Endothelial Cells in Young Men with Coronary Artery Disease. Atherosclerosis 129, 261–269. 10.1016/s0021-9150(96)06044-3 9105569

[B2] Amaroli A. Pasquale C. Zekiy A. Utyuzh A. Benedicenti S. Signore A. (2021). Photobiomodulation and Oxidative Stress: 980 Nm Diode Laser Light Regulates Mitochondrial Activity and Reactive Oxygen Species Production. Oxid Med. Cel Longev 2021, 6626286. 10.1155/2021/6626286 PMC7952159 33763170

[B3] Ash C. Dubec M. Donne K. Bashford T. (2017). Effect of Wavelength and Beam Width on Penetration in Light-Tissue Interaction Using Computational Methods. Lasers Med. Sci. 32, 1909–1918. 10.1007/s10103-017-2317-4 28900751 PMC5653719

[B4] Buzinari T. C. de Moraes T. F. Cárnio E. C. Lopes L. A. Salgado H. C. Rodrigues G. J. (2020). Photobiomodulation Induces Hypotensive Effect in Spontaneously Hypertensive Rats. Lasers Med. Sci. 35, 567–572. 10.1007/s10103-019-02849-7 31396793

[B5] Caetano K. S. Frade M. A. C. Minatel D. G. Santana L. Á. Enwemeka C. S. (2009). Phototherapy Improves Healing of Chronic Venous Ulcers. Photomed. Laser Surg. 27, 111–118. 10.1089/pho.2008.2398 19196110

[B6] Colombo E. Signore A. Aicardi S. Zekiy A. Utyuzh A. Benedicenti S. (2021). Experimental and Clinical Applications of Red and Near-Infrared Photobiomodulation on Endothelial Dysfunction: A Review. Biomedicines 9, 274. 10.3390/biomedicines9030274 33803396 PMC7998572

[B7] Desmet K. D. Paz D. A. Corry J. J. Eells J. T. Wong-Riley M. T. T. Henry M. M. (2006). Clinical and Experimental Applications of NIR-LED Photobiomodulation. Photomed. Laser Surg. 24, 121–128. 10.1089/pho.2006.24.121 16706690

[B8] Feelisch M. Rassaf T. Mnaimneh S. Singh N. Bryan N. S. Jourd′Heuil D. (2002). Concomitant S-, N-, and Heme-Nitros(yl)ation in Biological Tissues and Fluids: Implications for the Fate of NO *In Vivo* . FASEB J. 16, 1775–1785. 10.1096/fj.02-0363com 12409320

[B9] Freitas L. F. Hamblin M. R. (2016). Proposed Mechanisms of Photobiomodulation or Low-Level Light Therapy. IEEE J. Sel Top. Quan. Electron 22, 348–364. 10.1109/JSTQE.2016.2561201 PMC5215870 28070154

[B10] Furchgott R. F. Ehrreich S. J. Greenblatt E. (1961). The Photoactivated Relaxation of Smooth Muscle of Rabbit Aorta. J. Gen. Phys. 44, 499–419. 10.1085/jgp.44.3.499 PMC2195116 13702637

[B11] George S. Hamblin M. R. Abrahamse H. (2018). Effect of Red Light and Near Infrared Laser on the Generation of Reactive Oxygen Species in Primary Dermal Fibroblasts. J. Photochem. Photobiol. B 188, 60–68. 10.1016/j.jphotobiol.2018.09.004 30216761 PMC6214457

[B12] Hamblin M. R. (2016). Shining Light on the Head: Photobiomodulation for Brain Disorders. BBA Clin. 6, 113–124. 10.1016/j.bbacli.2016.09.002 27752476 PMC5066074

[B13] Holm S. (1979). A Simple Sequentially Rejective Multiple Test Procedure. Scand. J. Stat. 6, 65–70.

[B14] Homer K. L. Wanstall J. C. (2002). Inhibition of Rat Platelet Aggregation by the Diazeniumdiolate Nitric Oxide Donor MAHMA NONOate. Br. J Pharmacol 137, 1071–1081. 10.1038/sj.bjp.0704971 12429580 PMC1573589

[B15] Ieda N. Hotta Y. Yamauchi A. Nishikawa A. Sasamori T. Saitoh D. (2020). Development of a Red-Light-Controllable Nitric Oxide Releaser to Control Smooth Muscle Relaxation *In Vivo* . ACS Chem. Biol. 15, 2958–2965. 10.1021/acschembio.0c00601 33166443

[B16] Karu T. I. (2010). Multiple Roles of Cytochrome C Oxidase in Mammalian Cells under Action of Red and IR-A Radiation. IUBMB Life 62, 607–610. 10.1002/iub.359 20681024

[B17] Karu T. I. Pyatibrat L. V. Afanasyeva N. I. (2005). Cellular Effects of Low Power Laser Therapy Can Be Mediated by Nitric Oxide. Lasers Surg. Med. 36, 307–314. 10.1002/lsm.20148 15739174

[B18] Karu T. (2010). Mitochondrial Mechanisms of Photobiomodulation in Context of New Data about Multiple Roles of ATP. Photomed. Laser Surg. 28, 159–160. 10.1089/pho.2010.2789 20374017

[B19] Karu T. (1999). Primary and Secondary Mechanisms of Action of Visible to Near-IR Radiation on Cells. J. Photochem. Photobiol. B: Biol. 49, 1–17. 10.1016/s1011-1344(98)00219-x 10365442

[B20] Keszler A. Lindemer B. Hogg N. Lohr N. L. (2019). Ascorbate Attenuates Red Light Mediated Vasodilation: Potential Role of S-Nitrosothiols. Redox Biol. 20, 13–18. 10.1016/j.redox.2018.09.008 30261342 PMC6156744

[B21] Keszler A. Lindemer B. Hogg N. Weihrauch D. Lohr N. L. (2018). Wavelength-Dependence of Vasodilation and NO Release from S-Nitrosothiols and Dinitrosyl Iron Complexes by Far Red/Near Infrared Light. Arch. Biochem. Biophys. 649, 47–52. 10.1016/j.abb.2018.05.006 29752896

[B22] Keszler A. Lindemer B. Weihrauch D. Jones D. Hogg N. Lohr N. L. (2017). Red/Near Infrared Light Stimulates Release of an Endothelium Dependent Vasodilator and Rescues Vascular Dysfunction in a Diabetes Model. Free Radic. Biol. Med. 113, 157–164. 10.1016/j.freeradbiomed.2017.09.012 28935419 PMC5699925

[B23] Kozlov A. V. Staniek K. Nohl H. (1999). Nitrite Reductase Activity Is a Novel Function of Mammalian Mitochondria. FEBS Lett. 454, 127–130. 10.1016/s0014-5793(99)00788-7 10413109

[B24] Liang K.-Y. Zeger S. L. (1986). Longitudinal Data Analysis Using Generalized Linear Models. Biometrika 73, 13–22. 10.1093/biomet/73.1.13

[B25] Liu T. Schroeder H. J. Wilson S. M. Terry M. H. Romero M. Longo L. D. (2016). Local and Systemic Vasodilatory Effects of Low Molecular Weight S-Nitrosothiols. Free Radic. Biol. Med. 91, 215–223. 10.1016/j.freeradbiomed.2015.12.009 26686469 PMC4761500

[B26] Lohr N. L. Keszler A. Pratt P. Bienengraber M. Warltier D. C. Hogg N. (2009). Enhancement of Nitric Oxide Release from Nitrosyl Hemoglobin and Nitrosyl Myoglobin by Red/Near Infrared Radiation: Potential Role in Cardioprotection. J. Mol. Cell Cardiol. 47, 256–263. 10.1016/j.yjmcc.2009.03.009 19328206 PMC4329292

[B27] Lohr N. L. Ninomiya J. T. Warltier D. C. Weihrauch D. (2013). Far Red/Near Infrared Light Treatment Promotes Femoral Artery Collateralization in the Ischemic Hindlimb. J. Mol. Cel Cardiol 62, 36–42. 10.1016/j.yjmcc.2013.05.007 PMC3747970 23702287

[B28] Lundberg J. O. Gladwin M. T. Ahluwalia A. Benjamin N. Bryan N. S. Butler A. (2009). Nitrate and Nitrite in Biology, Nutrition and Therapeutics. Nat. Chem. Biol. 5, 865–869. 10.1038/nchembio.260 19915529 PMC4038383

[B29] Machha A. Schechter A. N. (2011). Dietary Nitrite and Nitrate: A Review of Potential Mechanisms of Cardiovascular Benefits. Eur. J. Nutr. 50, 293–303. 10.1007/s00394-011-0192-5 21626413 PMC3489477

[B30] Matsushita K. Sang Y. Ning H. Ballew S. H. Chow E. K. Grams M. E. (2019). Lifetime Risk of Lower‐Extremity Peripheral Artery Disease Defined by Ankle‐Brachial Index in the United States. Jaha 8, e012177. 10.1161/JAHA.119.012177 31500474 PMC6818002

[B31] Miller M. R. Megson I. L. Roseberry M. J. Mazzei F. A. Butler A. R. Webb D. J. (2000). Novel S-Nitrosothiols Do Not Engender Vascular Tolerance and Remain Effective in Glyceryl Trinitrate-Tolerant Rat Femoral Arteries. Eur. J. Pharmacol. 403, 111–119. 10.1016/s0014-2999(00)00572-0 10969151

[B32] Mitchell U. H. Mack G. L. (2013). Low-Level Laser Treatment with Near-Infrared Light Increases Venous Nitric Oxide Levels Acutely. Am. J. Phys. Med. Rehabil. 92, 151–156. 10.1097/phm.0b013e318269d70a 23334615

[B33] Mohler E. R. Hiatt W. R. Gornik H. L. Kevil C. G. Quyyumi A. Haynes W. G. (2014). Sodium Nitrite in Patients with Peripheral Artery Disease and Diabetes Mellitus: Safety, Walking Distance and Endothelial Function. Vasc. Med. 19, 9–17. 10.1177/1358863x13515043 24363302

[B34] Plass C. A. Loew H. G. Podesser B. K. Prusa A. M. (2012). Light-Induced Vasodilation of Coronary Arteries and its Possible Clinical Implication. Ann. Thorac. Surg. 93, 1181–1186. 10.1016/j.athoracsur.2011.12.062 22381453

[B35] Poyton R. O. Ball K. A. (2011). Therapeutic Photobiomodulation: Nitric Oxide and a Novel Function of Mitochondrial Cytochrome C Oxidase. Discov. Med. 11, 154–159. 21356170

[B36] Rodriguez J. Maloney R. E. Rassaf T. Bryan N. S. Feelisch M. (2003). Chemical Nature of Nitric Oxide Storage Forms in Rat Vascular Tissue. Proc. Natl. Acad. Sci. U.S.A. 100, 336–341. 10.1073/pnas.0234600100 12502793 PMC140970

[B37] Samouilov A. Zweier J. L. (1998). Development of Chemiluminescence-Based Methods for Specific Quantitation of Nitrosylated Thiols. Anal. Biochem. 258, 322–330. 10.1006/abio.1998.2609 9570848

[B38] Shaffer J. P. (1986). Modified Sequentially Rejective Multiple Test Procedures. J. Am. Stat. Assoc. 81, 826–831. 10.1080/01621459.1986.10478341

[B39] Shiva S. Gladwin M. T. (2009). Shining a Light on Tissue NO Stores: Near Infrared Release of NO from Nitrite and Nitrosylated Hemes. J. Mol. Cell Cardiol. 46, 1–3. 10.1016/j.yjmcc.2008.10.005 18992252

[B40] Wajih N. Alipour E. Rigal F. Zhu J. Perlegas A. Caudell D. L. (2021). Effects of Nitrite and Far-Red Light on Coagulation. Nitric Oxide 107, 11–18. 10.1016/j.niox.2020.11.005 33271226 PMC7855911

[B41] Wajih N. Basu S. Ucer K. B. Rigal F. Shakya A. Rahbar E. (2019). Erythrocytic Bioactivation of Nitrite and its Potentiation by Far-Red Light. Redox Biol. 20, 442–450. 10.1016/j.redox.2018.11.001 30423533 PMC6230921

[B42] Weihrauch D. Keszler A. Lindemer B. Krolikowski J. Lohr N. L. (2021). Red Light Stimulates Vasodilation through Extracellular Vesicle Trafficking. J. Photochem. Photobiol. B: Biol. 220, 112212. 10.1016/j.jphotobiol.2021.112212 PMC8240139 34049180

[B43] Wong-Riley M. T. T. Liang H. L. Eells J. T. Chance B. Henry M. M. Buchmann E. (2005). Photobiomodulation Directly Benefits Primary Neurons Functionally Inactivated by Toxins. Role of Cytochrome C Oxidase. J. Biol. Chem. 280, 4761–4771. 10.1074/jbc.m409650200 15557336

[B44] Yang Y. Tang G. Yan J. Park B. Hoffman A. Tie G. (2008). Cellular and Molecular Mechanism Regulating Blood Flow Recovery in Acute versus Gradual Femoral Artery Occlusion are Distinct in the Mouse. J. Vasc. Surg. 48, 1546–1558. 10.1016/j.jvs.2008.07.063 19118738 PMC2791875

[B45] Zweier J. L. Ilangovan G. (2020). Regulation of Nitric Oxide Metabolism and Vascular Tone by Cytoglobin. Antiox Redox Signaling 32, 1172–1187. 10.1089/ars.2019.7881 PMC7196366 31880165

