# Supplementary material for: In Vivo Characterization of a Red Light-Activated Vasodilation: A Photobiomodulation Study
Source: Front Physiol. 2022 May 2;13:880158. doi: 10.3389/fphys.2022.880158 (PMC9108481; doi:10.3389/fphys.2022.880158)
Supplement: Supplementary file 1 [file Table1.DOCX]

| **Table S1. Figure 1B sample size** | | | | |
| --- | --- | --- | --- | --- |
| Data set | Control | 25 mW/cm^2^ | 50 mW/cm^2^ | 100 mW/cm^2^ |
| 0 min | 19 |  |  |  |
| 5 min |  | 8 | 8 | 8 |
| 10 min |  | 8 | 8 | 8 |
| 15 min |  | 3 | 3 | 3 |

| **Table S2. Figure 2A sample size** | | | | |
| --- | --- | --- | --- | --- |
| Data set | Control | 25 mW/cm^2^ | 50 mW/cm^2^ | 100 mW/cm^2^ |
| 0 min | 15 |  |  |  |
| 5 min |  | 6 | 6 | 5 |
| 10 min |  | 5 | 4 | 5 |
| 15 min |  | 7 | 6 | 6 |

| **Table S3. Figure 2B sample size** | | | | |
| --- | --- | --- | --- | --- |
| Data set | Control | 25 mW/cm^2^ | 50 mW/cm^2^ | 100 mW/cm^2^ |
| 0 min | 9 |  |  |  |
| 5 min |  | 3 | 3 | 3 |
| 10 min |  | 3 | 3 | 3 |
| 15 min |  | 3 | 3 | 3 |

| **Table S4. Figure 2C sample size** | | | | |
| --- | --- | --- | --- | --- |
| Data set | Control | 25 mW/cm^2^ | 50 mW/cm^2^ | 100 mW/cm^2^ |
| 0 min | 9 |  |  |  |
| 5 min |  | 5 | 5 | 5 |
| 10 min |  | 5 | 5 | 5 |
| 15 min |  | 5 | 5 | 5 |

| **Table S5. Figure 3B sample size** | | | | |  |
| --- | --- | --- | --- | --- | --- |
| Data set | Control | Light | 5 min | 10 min | 30 min |
| 0 min | 8 | 7 | 8 | 7 | 8 |

| **Table S6. Figure 3C sample size** | | | | |  |
| --- | --- | --- | --- | --- | --- |
| Data set | Control | Light | 5 min | 10 min | 30 min |
| 0 min | 7 | 7 | 8 | 8 | 8 |

**Figure 3A**: N=8

**Figure 4**: N=8

**Figure 5D**: N=10

**Figure 6A-C**: N=10
