# Supplementary figures and images for: In Vivo Characterization of a Red Light-Activated Vasodilation: A Photobiomodulation Study
Source: Front Physiol. 2022 May 2;13:880158. doi: 10.3389/fphys.2022.880158 (PMC9108481; doi:10.3389/fphys.2022.880158)

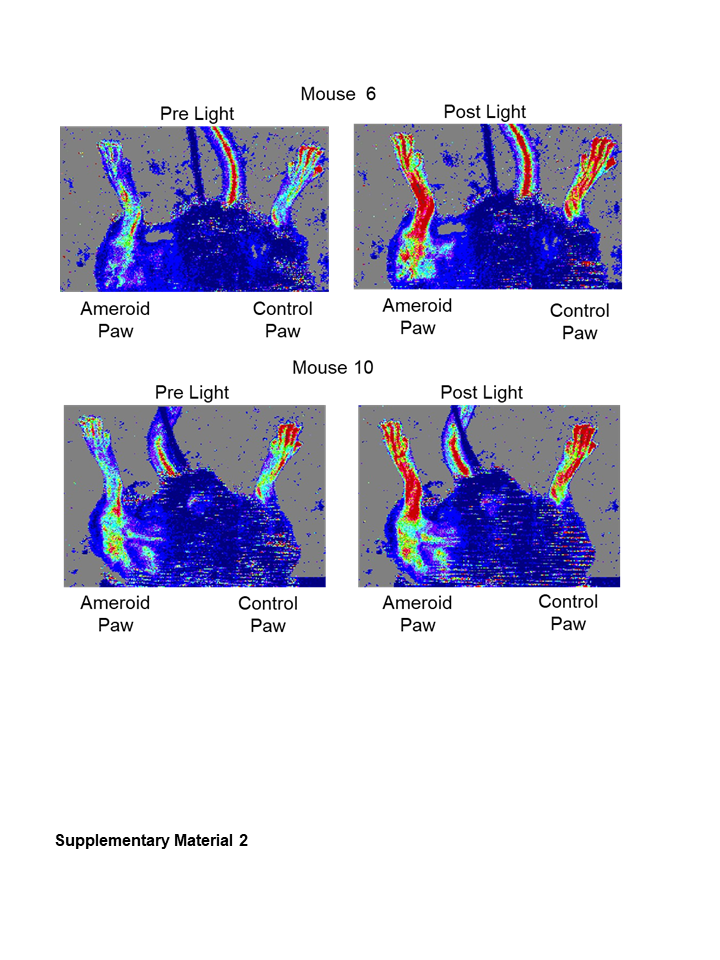

Supplement: Supplementary file 2 [file Image1.TIF]
